# Supplementary material for: Establishment and validation of a heart failure risk prediction model for elderly patients after coronary rotational atherectomy based on machine learning
Source: PeerJ. 2024 Jan 31;12:e16867. doi: 10.7717/peerj.16867 (PMC10838101; doi:10.7717/peerj.16867)
Supplement: Supplemental Information 3 [file peerj-12-16867-s003.docx]

**Results of Sensitivity Analysis**

**1 Missing value statistics results**

| **Variables** | **Total** | **Number of missing cases** | **Number of missing cases as a percentage of total** |
| --- | --- | --- | --- |
| **Diabetes** | **303** | **21** | **6.93%** |
| **Cerebrovascular disease** | **303** | **15** | **4.95%** |
| **Chronic renal insufficiency** | **303** | **31** | **10.23%** |
| **HbA1c** | **303** | **8** | **2.64%** |
| **Preoperative Troponin** | **303** | **11** | **3.63%** |
| **Blood cholesterol** | **303** | **13** | **4.29%** |
| **LDL-C** | **303** | **16** | **5.28%** |
| **VLDL-C** | **303** | **35** | **11.55%** |

**2 Results(Results of analyses without samples treated for missing values and sample equalization)**

**2.1 Comparison of Clinical Data of Patients**

**Among the 303 study subjects**, **53 patients** occured postoperative heart failure, with a heart failure incidence rate of **17.49%**. Compared with the non heart failure group, the heart failure group has a higher proportion of **chronic renal insufficiency, a lower LVEF, and higher levels of NT-proBNP, serum creatinine, fasting blood glucose, blood cholesterol, triglyceride, and low-density lipoprotein cholesterol, with statistically significant differences (all P<0.05)**. as shown in Table1.

**Table 1. Comparison of clinical data between two groups of patients**

| Vriables | Missing | Category | Total (n=303) | Non heart failure group(n=250) | Heart failure group (n=53) | χ^2^/z | P |
| --- | --- | --- | --- | --- | --- | --- | --- |
| Chronic renal insufficiency ,n(%) | 31 | No | 260(95.588) | 216(97.297) | 44(88.000) | 8.365^a^ | 0.004 |
|  |  | Yes | 12(4.412) | 6(2.703) | 6(12.000) |  |  |
| Ischemic cardiomyopathy ,n(%) | 0 | No | 286(94.389) | 238(95.200) | 48(90.566) | 1.773^a^ | 0.183 |
|  |  | Yes | 17(5.611) | 12(4.800) | 5(9.434) |  |  |
| Cerebrovascular disease ,n(%) | 15 | No | 237(82.292) | 193(81.780) | 44(84.615) | 0.235^a^ | 0.628 |
|  |  | Yes | 51(17.708) | 43(18.220) | 8(15.385) |  |  |
| Diabetes ,n(%) | 21 | No | 191(67.730) | 160(68.376) | 31(64.583) | 0.262^a^ | 0.609 |
|  |  | Yes | 91(32.270) | 74(31.624) | 17(35.417) |  |  |
| Hypertension ,n(%) | 0 | No | 130(42.904) | 110(44.000) | 20(37.736) | 0.700^a^ | 0.403 |
|  |  | Yes | 173(57.096) | 140(56.000) | 33(62.264) |  |  |
| Gender ,n(%) | 0 | Male | 174(57.426) | 140(56.000) | 34(64.151) | 1.188^a^ | 0.276 |
|  |  | Female | 129(42.574) | 110(44.000) | 19(35.849) |  |  |
| Age ,median[IQR] | 0 | nan | 72.000[67.000,77.000] | 72.000[67.000,76.000] | 72.000[67.000,77.000] | -0.917^b^ | 0.359 |
| LVEF ,median[IQR] | 0 | nan | 57.000[47.000,65.000] | 58.000[48.000,65.000] | 48.000[35.000,61.000] | 3.619^b^ | <0.001 |
| Preoperative CK-MB ,median[IQR] | 0 | nan | 16.000[12.000,24.000] | 15.000[12.000,24.000] | 17.000[14.000,23.000] | -1.867^b^ | 0.062 |
| Preoperative Troponin ,median[IQR] | 11 | nan | 0.100[0.010,0.650] | 0.100[0.010,0.650] | 0.070[0.010,0.640] | -0.266^b^ | 0.784 |
| NT-proBNP ,median[IQR] | 0 | nan | 1442.000[408.000,2856.000] | 1223.000[290.000,2533.000] | 3471.000[1897.000,6987.000] | -5.909^b^ | <0.001 |
| Serum creatinine ,median[IQR] | 0 | nan | 74.000[62.000,93.000] | 73.000[60.000,90.000] | 90.000[70.000,136.000] | -4.158^b^ | <0.001 |
| Fasting blood glucose ,median[IQR] | 0 | nan | 5.510[4.790,7.170] | 5.400[4.750,6.850] | 7.100[5.360,9.130] | -3.984^b^ | <0.001 |
| HbA1c ,median[IQR] | 8 | nan | 6.800[5.900,7.900] | 6.800[5.900,7.700] | 7.000[5.900,8.680] | -1.332^b^ | 0.183 |
| Hemoglobin ,median[IQR] | 0 | nan | 121.000[110.000,131.000] | 122.000[110.000,132.000] | 112.000[108.000,130.000] | 1.576^b^ | 0.115 |
| Blood cholesterol ,median[IQR] | 13 | nan | 3.740[3.120,4.520] | 3.660[3.100,4.440] | 4.380[3.320,6.210] | -3.200^b^ | 0.001 |
| Triglyceride ,median[IQR] | 0 | nan | 1.220[0.950,1.610] | 1.170[0.950,1.570] | 1.390[1.120,2.420] | -2.410^b^ | 0.016 |
| LDLC ,median[IQR] | 16 | nan | 1.880[1.490,2.440] | 1.870[1.480,2.330] | 2.120[1.560,2.680] | -2.156^b^ | 0.031 |
| V-LDLC ,median[IQR] | 35 | nan | 0.840[0.680,1.040] | 0.830[0.660,1.020] | 0.870[0.760,1.060] | -1.428^b^ | 0.154 |

Note: a. Pearson Chi-squared test, b. Mann Whitney rank sum test. LVEF, left ventricular Ejection fraction; NT-proBNP, N-terminal pro brain natriuretic peptide; CK-MB, creatine kinase isoenzymes; HbA1c, Glycated hemoglobin.

**2.2 Feature variable screening results**

This study randomly divided the data of 303 patients into a training set and a testing set in a 7:3 ratio, consisting of 212 and 91 patients, respectively. There was no statistically significant difference in clinical data between the two groups of patients (P>0.05), indicating that the two datasets were homogeneous and comparable. In the training set, the LASSO regression was used to screen the characteristic variables of 19 indicators, and the variable of non-zero regression coefficient corresponding to the Lambda coefficient of the minimum distance Standard error was selected as the characteristic variable through 10 times cross validation. The LASSO regression results show that the Lambda coefficient of the minimum distance Standard error(Lambda.1se) is **0.084**, and the corresponding characteristic variables include **blood cholesterol, fasting blood glucose, chronic renal insufficiency, NT-proBNP and LVEF**; The Lambda coefficient of Minimum mean square error(Lambda.min) is **0.033**, and the corresponding characteristic variables include **triglyceride, blood cholesterol, fasting blood glucose, creatinine, NT-proBNP, LVEF, chronic renal insufficiency**. As shown in Figure1.

**A B**


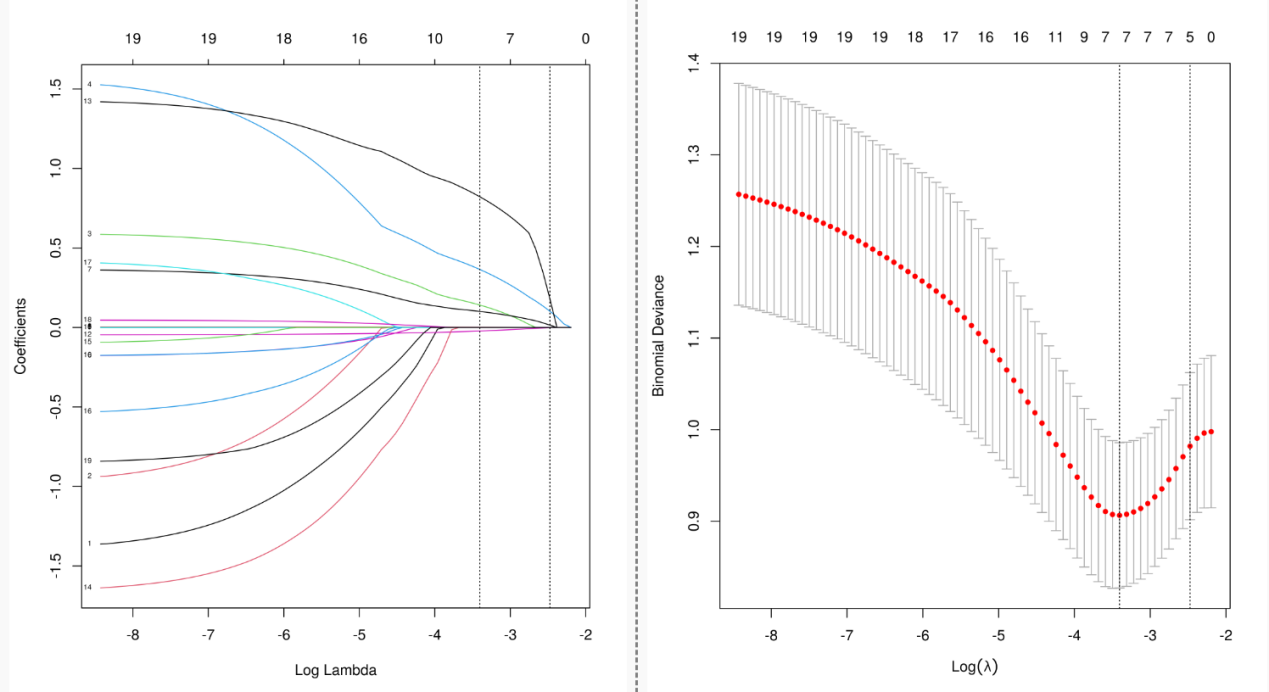


**Figure 1. Lasso regression analysis results**

Note: A. Lasso regression coefficient diagram; B. Lasso regression cross validation statistical chart. The two vertical dashed lines in the chart represent the logarithmic Lambda coefficient of the Minimum mean square error (dashed line on the left) and the logarithmic Lambda coefficient of the Standard error of the minimum distance (dashed line on the right).

**2.3 Model construction**

In the training set, four algorithms including LR, XGboost, LightGBM and SVM, were used to build a machine learning prediction model including 5 variables( **blood cholesterol, fasting blood glucose, chronic renal insufficiency, NT-proBNP and LVEF**) based on the Lambda.1se results, and 10 times cross validation was used to internally verify the built prediction model. The results showed that the XGBoost model had the highest AUC in the training set and the internal validation set, suggesting that XGBoost was the optimal model. As shown in Table 2 and Figure 2.

**Table 2. Comparison Results of Multiple Models**

| Classification model | AUC | Cutoff | Accuracy | Sensitivity | Specificity | Positive predictive value | Negative predictive value | F1 Score |
| --- | --- | --- | --- | --- | --- | --- | --- | --- |
| **Training Set** |  |  |  |  |  |  |  |  |
| XGBoost | 1.000 | 0.757 | 0.994 | 1.000 | 1.000 | 1.000 | 0.993 | 1.000 |
| Logistic | 0.909 | 0.224 | 0.864 | 0.801 | 0.884 | 0.566 | 0.951 | 0.660 |
| LightGBM | 1.000 | 0.793 | 0.994 | 1.000 | 1.000 | 1.000 | 0.993 | 1.000 |
| SVM | 0.702 | 0.240 | 0.752 | 0.636 | 0.781 | NA | 0.919 | NA |
| **Internal validation set** |  |  |  |  |  |  |  |  |
| XGBoost | 0.907 | 0.757 | 0.877 | 0.967 | 0.827 | Na | 0.888 | Na |
| Logistic | 0.904 | 0.224 | 0.866 | 0.967 | 0.853 | 0.693 | 0.954 | 0.773 |
| LightGBM | 0.890 | 0.793 | 0.866 | 0.967 | 0.800 | Na | 0.881 | Na |
| SVM | 0.658 | 0.240 | 0.699 | 0.767 | 0.740 | Na | 0.889 | Na |

Note: NA, Cannot be calculated.

**A B**


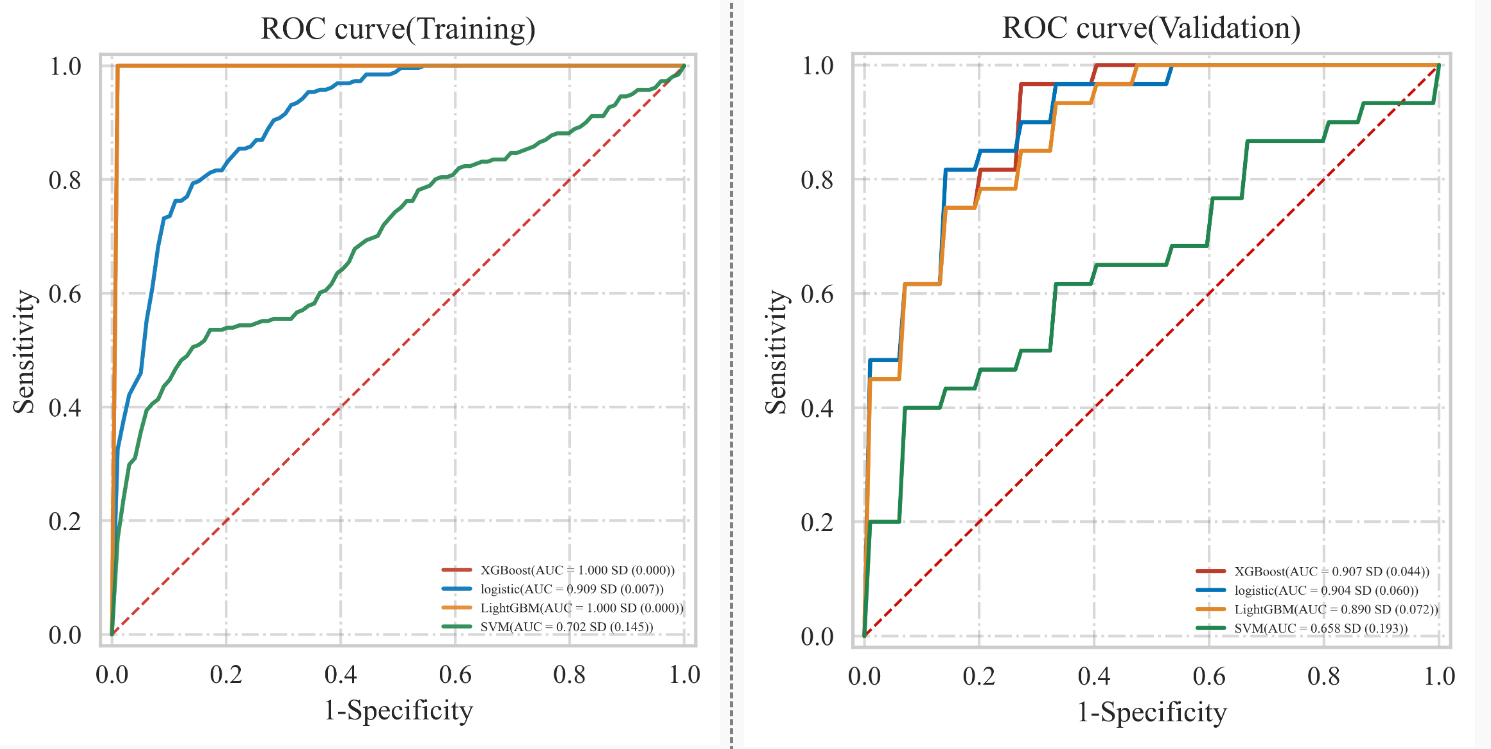


**Figure 2. ROC curve of multiple models**

Note: A. ROC curve in training set; B. ROC curve in the internal validation set.

**2.4 Model Performance Evaluation**

Testing the predictive ability of the XGBoost algorithm on the risk of heart failure in patients after CRA in the validation set. The results showed that the AUC of the XGBoost model in the validation set was **0.809**, the prediction accuracy was **0.800**, the sensitivity was **0.850**, the specificity was **0.717**, the positive predictive value was **0.833**, the negative predictive value was **0.797**, and the F1 score was **0.842**. See Figure3 for the Receiver operating characteristic of XGBoost model in the validation set.


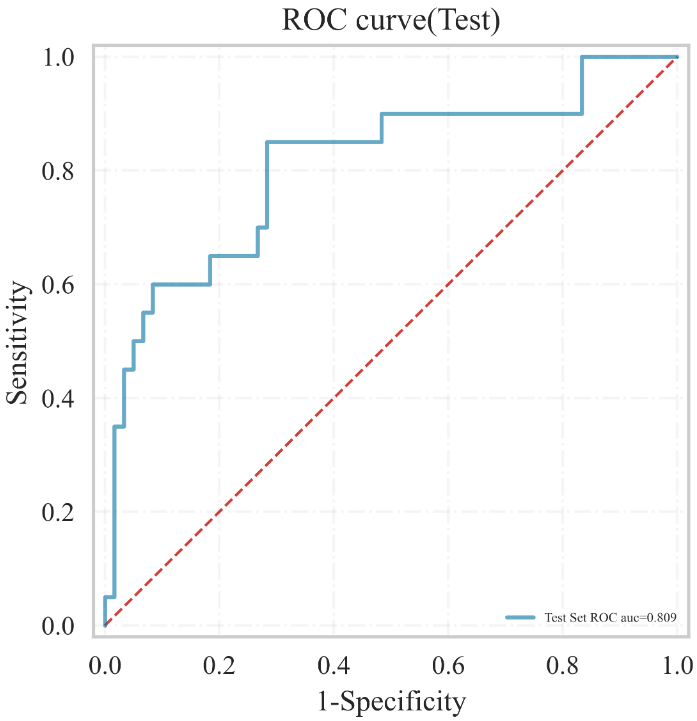


**Figure 3. XGBoost's ROC curve in the validation set**

**3 Results(Results of analyses with samples treated for missing values and without sample equalization, results of data analyses in the original manuscript)**

**3.1 Comparison of Clinical Data of Patients**

Among the **303 study subjects**, **53** patients occured postoperative heart failure, with a heart failure incidence rate of **17.49%**. Compared with the non heart failure group, the heart failure group has a higher proportion of **chronic renal insufficiency, a lower LVEF, and higher levels of NT-proBNP, serum creatinine, fasting blood glucose, blood cholesterol, triglyceride, and low-density lipoprotein cholesterol, with statistically significant differences (all P<0.05)**. as shown in Table1.

**Table 1. Comparison of clinical data between two groups of patients**

| Variables | Total (n=303) | Non heart failure group (n=250) | Heart failure group (n=53) | t/z | P |
| --- | --- | --- | --- | --- | --- |
| Gender, n (%) |  |  |  | 1.188^a^ | 0.276 |
| Male | 174 (57.43) | 140 (56.00) | 34 (64.15) |  |  |
| Female | 129 (42.57) | 110 (44.00) | 19 (35.85) |  |  |
| Combined with chronic renal insufficiency, n (%) | 14 (4.62) | 8 (3.20) | 6 (11.32) | 6.544^a^ | 0.011 |
| Combined with ischemic cardiomyopathy, n (%) | 17 (5.61) | 12 (4.80) | 5 (9.43) | 1.773^a^ | 0.183 |
| Combined with Cerebrovascular disease, n (%) | 60 (19.80) | 51 (20.40) | 9 (16.98) | 0.322^a^ | 0.570 |
| Combined with diabetes, n (%) | 99 (32.67) | 81 (32.40) | 18 (33.96) | 0.049^a^ | 0.826 |
| Concomitant with hypertension, n (%) | 173 (57.10) | 140 (56.00) | 33 (62.26) | 0.700^a^ | 0.403 |
| Age | 72.00 (67.00, 77.00) | 72.00 (67.00, 76.00) | 72.00 (67.00, 77.00) | -0.917^b^ | 0.359 |
| LVEF | 57.00 (47.00,65.00) | 58.00 (48.00,65.00) | 48.00 (35.00,61.00) | 3.619^b^ | <0.001 |
| Preoperative CK-MB | 16.00 (12.00,24.00) | 15.00 (12.00,24.00) | 17.00 (14.00, 23.00) | -1.867^b^ | 0.062 |
| Preoperative Troponin | 0.12 (0.01,0.65) | 0.13 (0.01,0.65) | 0.08 (0.01,0.64) | -0.280^b^ | 0.773 |
| NT-proBNP | 1442.00 (408.002856.00) | 1223.00 (290.002533.00) | 3471.00 (1897.006987.00) | -5.909^b^ | <0.001 |
| Serum creatinine | 74.00 (62.00,93.00) | 73.00 (60.00, 90.00) | 90.00 (70.00136.00) | -4.158^b^ | <0.001 |
| Fasting blood glucose | 5.51 (4.79,7.17) | 5.40 (4.75,6.85) | 7.10 (5.36,9.13) | -3.984^b^ | <0.001 |
| HbA1c | 6.80 (5.90,7.90) | 6.80 (5.90,7.79) | 7.00 (5.90, 8.68) | -1.245^b^ | 0.213 |
| hemoglobin | 121.00 (110.00131.00) | 122.00 (110.00132.00) | 112.00 (108.00130.00) | 1.576^b^ | 0.115 |
| Blood cholesterol | 3.73 (3.10,4.49) | 3.65 (3.08, 4.37) | 4.36 (3.27, 6.21) | -3.127^b^ | 0.002 |
| Triglyceride | 1.22 (0.95,1.61) | 1.17 (0.95,1.57) | 1.39 (1.12,2.42) | -2.410^b^ | 0.016 |
| Low density lipoprotein cholesterol | 1.90 (1.49,2.44) | 1.87 (1.48,2.33) | 2.13 (1.58, 2.68) | -2.390^b^ | 0.017 |
| Very low-density lipoprotein cholesterol | 0.84 (0.68,1.02) | 0.83 (0.66,1.01) | 0.88 (0.76,1.06) | -1.827^b^ | 0.068 |

Note: a. Pearson Chi-squared test, b. Mann Whitney rank sum test. LVEF, left ventricular Ejection fraction; NT-proBNP, N-terminal pro brain natriuretic peptide; CK-MB, creatine kinase isoenzymes; HbA1c, Glycated hemoglobin.

**3.2 Feature variable screening results**

This study randomly divided the data of 303 patients into a training set and a testing set in a 7:3 ratio, consisting of 212 and 91 patients, respectively. There was no statistically significant difference in clinical data between the two groups of patients (P>0.05), indicating that the two datasets were homogeneous and comparable. In the training set, the LASSO regression was used to screen the characteristic variables of 19 indicators, and the variable of non-zero regression coefficient corresponding to the Lambda coefficient of the minimum distance Standard error was selected as the characteristic variable through 10 times cross validation. The LASSO regression results show that the Lambda coefficient of the minimum distance Standard error(Lambda.1se) is **0.07**, and the corresponding characteristic variables include **blood cholesterol, fasting blood glucose, serum creatinine, NT-proBNP and LVEF**; The Lambda coefficient of Minimum mean square error(Lambda.min) is **0.023**, and the corresponding characteristic variables include **triglyceride, blood cholesterol, fasting blood glucose, creatinine, NT-proBNP, LVEF, chronic renal insufficiency, age and gender**. As shown in Figure1.


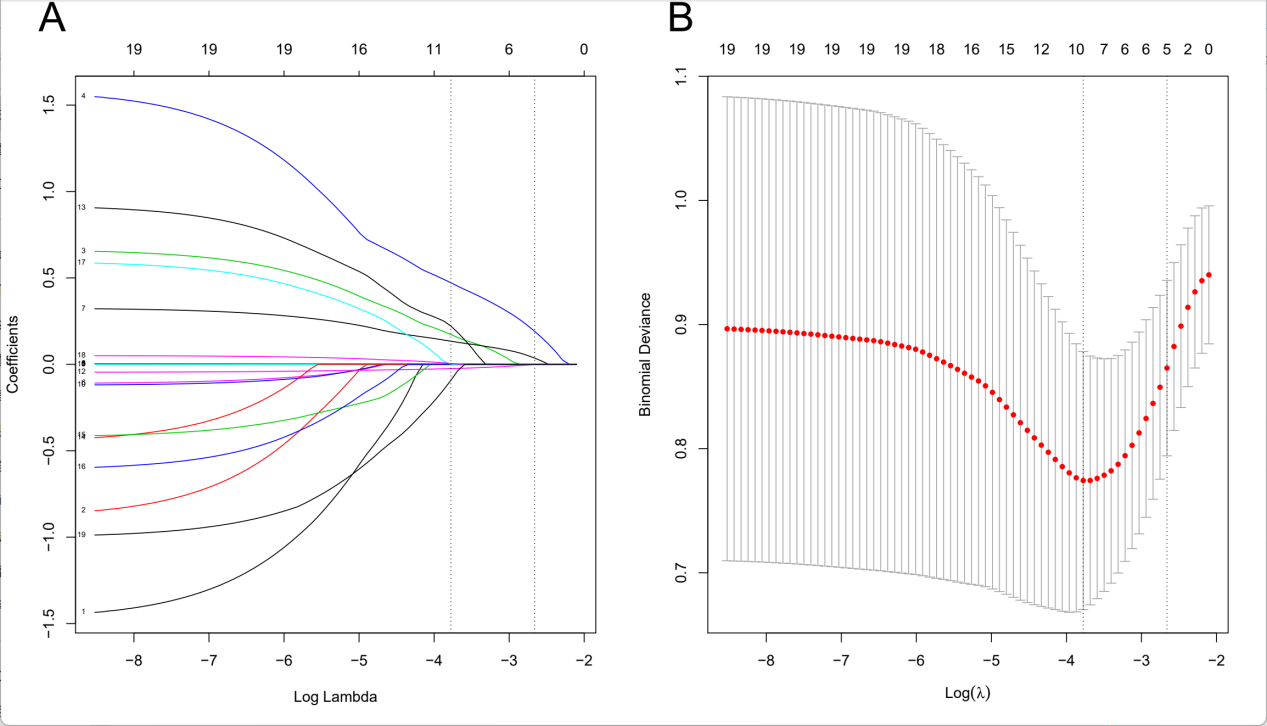


**Figure 1. Lasso regression analysis results**

Note: A. Lasso regression coefficient diagram; B. Lasso regression cross validation statistical chart. The two vertical dashed lines in the chart represent the logarithmic Lambda coefficient of the Minimum mean square error (dashed line on the left) and the logarithmic Lambda coefficient of the Standard error of the minimum distance (dashed line on the right).

**3.3 Model construction**

In the training set, four algorithms including LR, XGboost, LightGBM and SVM, were used to build a machine learning prediction model including 5 variables( **blood cholesterol, fasting blood glucose, serum creatinine, NT-proBNP and LVEF**) based on the Lambda.1se results, and 10 times cross validation was used to internally verify the built prediction model. The results showed that the XGBoost model had the highest AUC in the training set and the internal validation set, suggesting that XGBoost was the optimal model. As shown in Table 2 and Figure 2.

**Table 2. Comparison Results of Multiple Models**

| Classification model | AUC | Cutoff | Accuracy | Sensitivity | Specificity | Positive predictive value | Negative predictive value | F1 Score |
| --- | --- | --- | --- | --- | --- | --- | --- | --- |
| **Training Set** |  |  |  |  |  |  |  |  |
| XGBoost | 1.000 | 0.775 | 0.995 | 1.000 | 1.000 | 1.000 | 0.994 | 1.000 |
| Logistic | 0.891 | 0.128 | 0.790 | 0.879 | 0.780 | 0.417 | 0.966 | 0.560 |
| LightGBM | 1.000 | 0.841 | 0.995 | 1.000 | 1.000 | 1.000 | 0.994 | 1.000 |
| SVM | 0.681 | 0.123 | 0.838 | 0.511 | 0.902 | 0.466 | 0.906 | 0.486 |
| **Internal validation set** |  |  |  |  |  |  |  |  |
| XGBoost | 0.888 | 0.775 | 0.897 | 0.900 | 0.878 | NA | 0.900 | NA |
| Logistic | 0.865 | 0.128 | 0.760 | 0.933 | 0.828 | 0.401 | 0.949 | 0.554 |
| LightGBM | 0.884 | 0.841 | 0.882 | 0.867 | 0.867 | NA | 0.887 | NA |
| SVM | 0.618 | 0.123 | 0.812 | 0.708 | 0.783 | NA | 0.901 | NA |

Note: NA, Cannot be calculated.


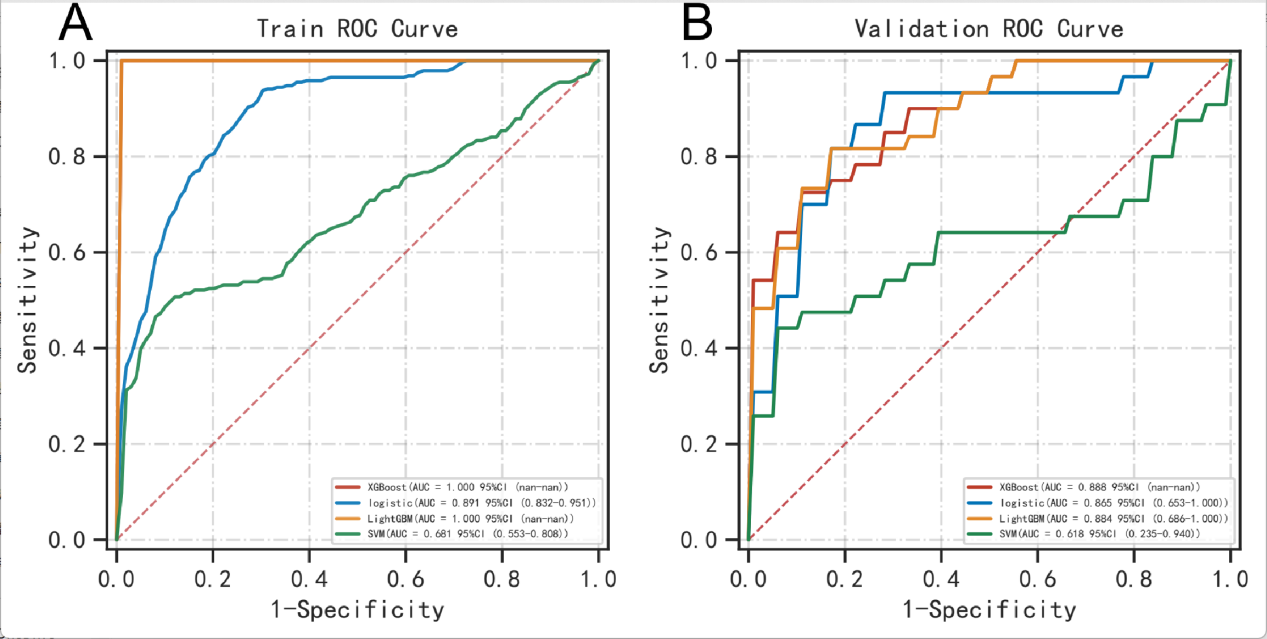


**Figure 2. ROC curve of multiple models**

Note: A. ROC curve in training set; B. ROC curve in the internal validation set.

**3.4 Model Performance Evaluation**

Testing the predictive ability of the XGBoost algorithm on the risk of heart failure in patients after CRA in the validation set. The results showed that the AUC of the XGBoost model in the validation set was 0.852, the prediction accuracy was 0.868, the sensitivity was 0.857, the specificity was 0.800, the positive predictive value was 0.909, the negative predictive value was 0.863, and the F1 score was 0.882. See Figure3 for the Receiver operating characteristic of XGBoost model in the validation set.


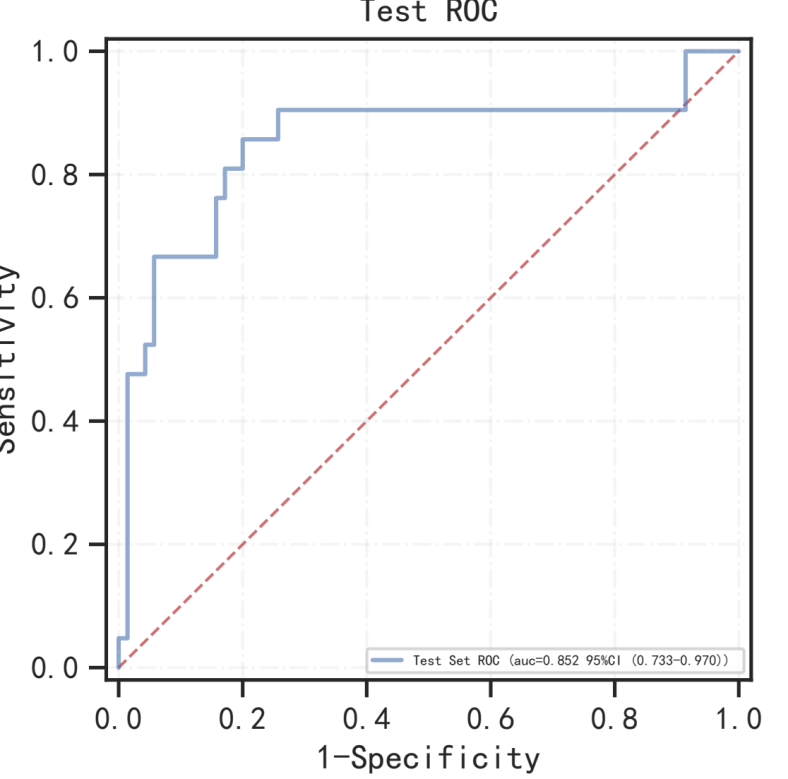


**Figure 3. XGBoost's ROC curve in the validation set**

**4 Results(Results of analyses with samples treated for missing values and sample equalization according to the “Reviewer 2’s suggestions” and “Reviewer 3’s suggestions”, results of data analyses in the revised manuscript)**

**4.1 Comparison of Clinical Data of Patients**

Among the **303 study subjects**, **53** patients occured postoperative heart failure, with a heart failure incidence rate of **17.49%**. Compared with the non heart failure group, the heart failure group has a higher proportion of **chronic renal insufficiency, a lower LVEF, and higher levels of NT-proBNP, serum creatinine, fasting blood glucose, blood cholesterol, triglyceride, and low-density lipoprotein cholesterol, with statistically significant differences (all P<0.05)**. as shown in Table1.

**Table 1. Comparison of clinical data between two groups of patients**

| Variables | Total (n=303) | Non heart failure group (n=250) | Heart failure group (n=53) | t/z | P |
| --- | --- | --- | --- | --- | --- |
| Gender, n (%) |  |  |  | 1.188^a^ | 0.276 |
| Male | 174 (57.43) | 140 (56.00) | 34 (64.15) |  |  |
| Female | 129 (42.57) | 110 (44.00) | 19 (35.85) |  |  |
| Combined with chronic renal insufficiency, n (%) | 14 (4.62) | 8 (3.20) | 6 (11.32) | 6.544^a^ | 0.011 |
| Combined with ischemic cardiomyopathy, n (%) | 17 (5.61) | 12 (4.80) | 5 (9.43) | 1.773^a^ | 0.183 |
| Combined with Cerebrovascular disease, n (%) | 60 (19.80) | 51 (20.40) | 9 (16.98) | 0.322^a^ | 0.570 |
| Combined with diabetes, n (%) | 99 (32.67) | 81 (32.40) | 18 (33.96) | 0.049^a^ | 0.826 |
| Concomitant with hypertension, n (%) | 173 (57.10) | 140 (56.00) | 33 (62.26) | 0.700^a^ | 0.403 |
| Age | 72.00 (67.00, 77.00) | 72.00 (67.00, 76.00) | 72.00 (67.00, 77.00) | -0.917^b^ | 0.359 |
| LVEF | 57.00 (47.00,65.00) | 58.00 (48.00,65.00) | 48.00 (35.00,61.00) | 3.619^b^ | <0.001 |
| Preoperative CK-MB | 16.00 (12.00,24.00) | 15.00 (12.00,24.00) | 17.00 (14.00, 23.00) | -1.867^b^ | 0.062 |
| Preoperative Troponin | 0.12 (0.01,0.65) | 0.13 (0.01,0.65) | 0.08 (0.01,0.64) | -0.280^b^ | 0.773 |
| NT-proBNP | 1442.00 (408.002856.00) | 1223.00 (290.002533.00) | 3471.00 (1897.006987.00) | -5.909^b^ | <0.001 |
| Serum creatinine | 74.00 (62.00,93.00) | 73.00 (60.00, 90.00) | 90.00 (70.00136.00) | -4.158^b^ | <0.001 |
| Fasting blood glucose | 5.51 (4.79,7.17) | 5.40 (4.75,6.85) | 7.10 (5.36,9.13) | -3.984^b^ | <0.001 |
| HbA1c | 6.80 (5.90,7.90) | 6.80 (5.90,7.79) | 7.00 (5.90, 8.68) | -1.245^b^ | 0.213 |
| hemoglobin | 121.00 (110.00131.00) | 122.00 (110.00132.00) | 112.00 (108.00130.00) | 1.576^b^ | 0.115 |
| Blood cholesterol | 3.73 (3.10,4.49) | 3.65 (3.08, 4.37) | 4.36 (3.27, 6.21) | -3.127^b^ | 0.002 |
| Triglyceride | 1.22 (0.95,1.61) | 1.17 (0.95,1.57) | 1.39 (1.12,2.42) | -2.410^b^ | 0.016 |
| Low density lipoprotein cholesterol | 1.90 (1.49,2.44) | 1.87 (1.48,2.33) | 2.13 (1.58, 2.68) | -2.390^b^ | 0.017 |
| Very low-density lipoprotein cholesterol | 0.84 (0.68,1.02) | 0.83 (0.66,1.01) | 0.88 (0.76,1.06) | -1.827^b^ | 0.068 |

Note: a. Pearson Chi-squared test, b. Mann Whitney rank sum test. LVEF, left ventricular Ejection fraction; NT-proBNP, N-terminal pro brain natriuretic peptide; CK-MB, creatine kinase isoenzymes; HbA1c, Glycated hemoglobin.

**4.2** **Comparison of training set and testing set**

After processing the missing values in the original data and addressing sample imbalance using Adaptive Synthetic Sampling (ADASYN) method , the final dataset consists of 502 samples: 250 negative samples (i.e., patients not suffering from heart failure) and 252 positive samples (i.e., patients with heart failure). This study randomly divided the data of 502 patients into a training set and a testing set in a 7:3 ratio, consisting of 351 and 151 patients, respectively. There was no statistically significant difference in clinical data between the two groups of patients (P>0.05), indicating that the two datasets were homogeneous and comparable. As shown in Table2.

**Table2.Comparison of training set and testing set**

| Variables | Category | Total (n=502) | Training set(n=351) | Testing set(n=151) | Χ^2^/z | P |
| --- | --- | --- | --- | --- | --- | --- |
| Heart failure ,n(%) | No | 250(49.80) | 176(50.14) | 74(49.01) | 0.054^a^ | 0.815 |
|  | Yes | 252(50.20) | 175(49.86) | 77(50.99) |  |  |
| Combined with Chronic renal insufficiency ,n(%) | No | 485(96.61) | 337(96.01) | 148(98.01) | 1.293^a^ | 0.255 |
|  | Yes | 17(3.39) | 14(3.99) | 3(1.99) |  |  |
| Combined with Ischemic cardiomyopathy ,n(%) | No | 481(95.82) | 337(96.01) | 144(95.36) | 0.110^a^ | 0.740 |
|  | Yes | 21(4.18) | 14(3.99) | 7(4.64) |  |  |
| Combined with Cerebrovascular disease ,n(%) | No | 435(86.65) | 307(87.46) | 128(84.77) | 0.664^a^ | 0.415 |
|  | Yes | 67(13.35) | 44(12.54) | 23(15.23) |  |  |
| Combined with Diabetes ,n(%) | No | 359(71.51) | 254(72.36) | 105(69.54) | 0.415^a^ | 0.520 |
|  | Yes | 143(28.49) | 97(27.64) | 46(30.46) |  |  |
| Combined with Hypertension ,n(%) | No | 245(48.80) | 172(49.00) | 73(48.34) | 0.018^a^ | 0.892 |
|  | Yes | 257(51.20) | 179(51.00) | 78(51.66) |  |  |
| Gender ,n(%) | Male | 340(67.73) | 244(69.52) | 96(63.58) | 1.704^a^ | 0.192 |
|  | Female | 162(32.27) | 107(30.48) | 55(36.42) |  |  |
| Age | / | 72.00(67.00,76.00) | 72.00(67.00,76.00) | 72.00(67.00,76.00) | -0.798^b^ | 0.424 |
| LVEF | / | 54.00(44.00,62.00) | 55.00(43.00,62.00) | 53.00(45.00,63.00) | -0.023^b^ | 0.982 |
| Preoperative CK-MB | / | 16.12(13.00,24.00) | 16.57(13.00,24.88) | 16.01(13.00,23.93) | 0.218^b^ | 0.827 |
| Preoperative Troponin | / | 0.14(0.01,0.65) | 0.10(0.01,0.64) | 0.23(0.01,0.65) | -1.317^b^ | 0.181 |
| NT-proBNP | / | 1880.00(643.00,3471.00) | 1858.00(676.00,3499.00) | 1897.00(641.00,2985.00) | 0.506^b^ | 0.613 |
| Serum creatinine | / | 82.00(66.66,105.00) | 81.79(68.04,105.00) | 82.00(64.00,105.00) | 0.339^b^ | 0.734 |
| Fasting blood glucose | / | 6.62(5.04,7.84) | 6.54(5.05,7.78) | 6.76(5.05,8.01) | -0.886^b^ | 0.376 |
| HbA1c | / | 6.98(5.92,8.10) | 6.98(5.90,8.05) | 7.00(6.03,8.16) | -0.704^b^ | 0.481 |
| Hemoglobin | / | 119.83(109.57,130.00) | 120.06(109.42,131.00) | 116.00(110.00,127.00) | 1.665^b^ | 0.096 |
| Blood cholesterol | / | 4.05(3.25,4.99) | 4.12(3.25,5.09) | 3.90(3.25,4.71) | 1.129^b^ | 0.259 |
| Triglyceride | / | 1.31(1.06,1.76) | 1.35(1.06,1.75) | 1.24(1.06,1.85) | 0.035^b^ | 0.973 |
| Low density lipoprotein cholesterol | / | 2.02(1.53,2.59) | 2.02(1.57,2.63) | 2.03(1.52,2.48) | 0.640^b^ | 0.523 |
| Very low-density lipoprotein cholesterol | / | 0.87(0.74,1.04) | 0.88(0.75,1.04) | 0.87(0.74,1.04) | 0.540^b^ | 0.590 |

Note: a. Pearson Chi-squared test, b. Mann Whitney rank sum test. LVEF, left ventricular Ejection fraction; NT-proBNP, N-terminal pro brain natriuretic peptide; CK-MB, creatine kinase isoenzymes; HbA1c, Glycated hemoglobin.

**4.3 Feature variable screening results**

In the training set, the LASSO regression was used to screen the characteristic variables of 19 indicators, and the variable of non-zero regression coefficient corresponding to the Lambda coefficient of the minimum distance Standard error was selected as the characteristic variable through 10 times cross validation. The LASSO regression results show that the Lambda coefficient of the minimum distance Standard error(Lambda.1se) is **0.037**, and the corresponding characteristic variables include **Triglyceride, blood cholesterol, fasting blood glucose, creatinine, NT-proBNP, LVEF, age** ; The Lambda coefficient of Minimum mean square error(Lambda.min) is **0.014**, and the corresponding characteristic variables were as same as the results of Lambda.1se, which include **VLDL-C, triglyceride, blood cholesterol, HbA1c, fasting blood glucose, creatinine, NT-proBNP, LVEF, chronic renal insufficiency, Ischemic cardiomyopathy,Cerebrovascular disease,Diabetes,Age, Gender**. As shown in Figure1.

**A B**


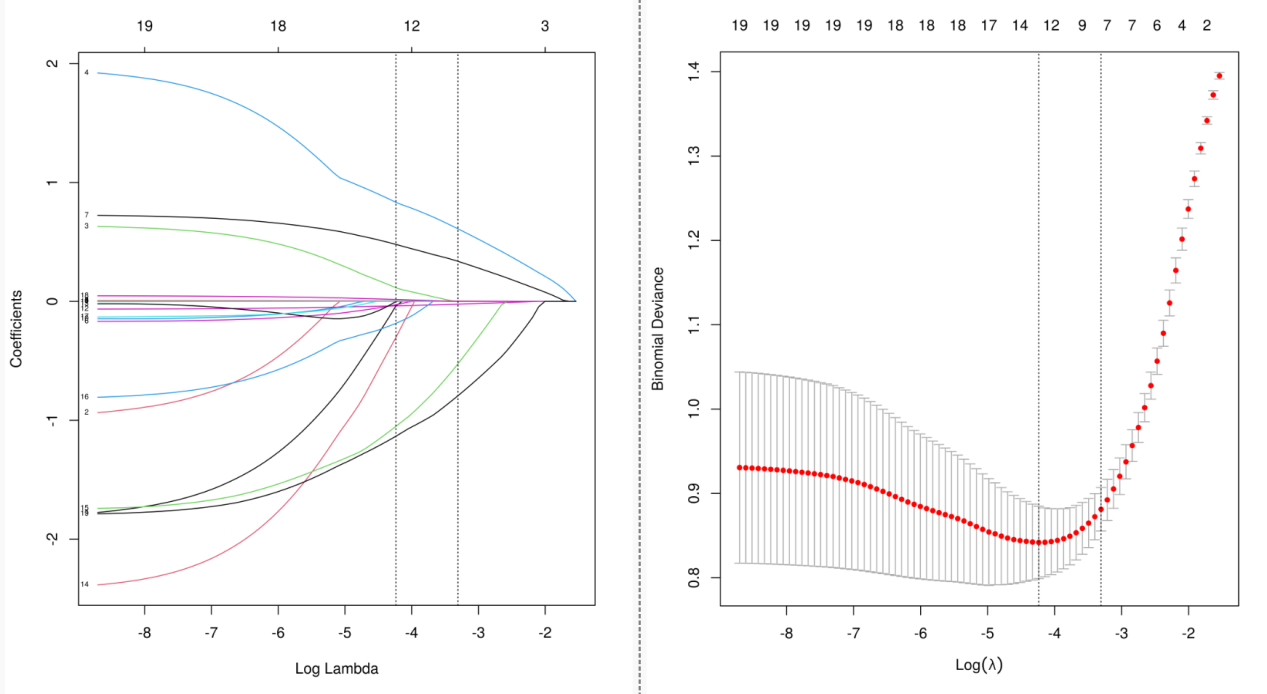


**Figure 1. Lasso regression analysis results**

Note: A. Lasso regression coefficient diagram; B. Lasso regression cross validation statistical chart. The two vertical dashed lines in the chart represent the logarithmic Lambda coefficient of the Minimum mean square error (dashed line on the left) and the logarithmic Lambda coefficient of the Standard error of the minimum distance (dashed line on the right).

**4.4 Model construction**

In the training set, four algorithms including LR, XGboost, LightGBM and SVM, were used to build a machine learning prediction model including 7 variables(**Triglyceride, blood cholesterol, fasting blood glucose, creatinine, NT-proBNP, LVEF, age**) based on the Lambda.1se results, and 10 times cross validation was used to internally verify the built prediction model. The results showed that the XGBoost model had the highest AUC in the training set and the internal validation set, suggesting that XGBoost was the optimal model. As shown in Table 3 and Figure 2.

**Table 3. Comparison Results of Multiple Models**

| Classification model | AUC | Cutoff | Accuracy | Sensitivity | Specificity | Positive predictive value | Negative predictive value | F1 Score |
| --- | --- | --- | --- | --- | --- | --- | --- | --- |
| **Training Set** |  |  |  |  |  |  |  |  |
| XGBoost | 1.000 | 0.831 | 0.997 | 1.000 | 1.000 | 1.000 | 0.994 | 1.000 |
| Logistic | 0.872 | 0.475 | 0.808 | 0.845 | 0.778 | 0.790 | 0.830 | 0.817 |
| LightGBM | 1.000 | 0.890 | 0.997 | 1.000 | 1.000 | 1.000 | 0.994 | 1.000 |
| SVM | 0.699 | 0.463 | 0.686 | 0.690 | 0.690 | 0.686 | 0.686 | 0.688 |
| **Internal validation set** |  |  |  |  |  |  |  |  |
| XGBoost | 0.972 | 0.831 | 0.889 | 0.978 | 0.909 | 0.929 | 0.864 | 0.951 |
| Logistic | 0.863 | 0.475 | 0.801 | 0.813 | 0.841 | 0.785 | 0.825 | 0.796 |
| LightGBM | 0.963 | 0.890 | 0.863 | 0.977 | 0.915 | 0.940 | 0.823 | 0.957 |
| SVM | 0.696 | 0.463 | 0.678 | 0.733 | 0.689 | 0.684 | 0.682 | 0.698 |

**A B**


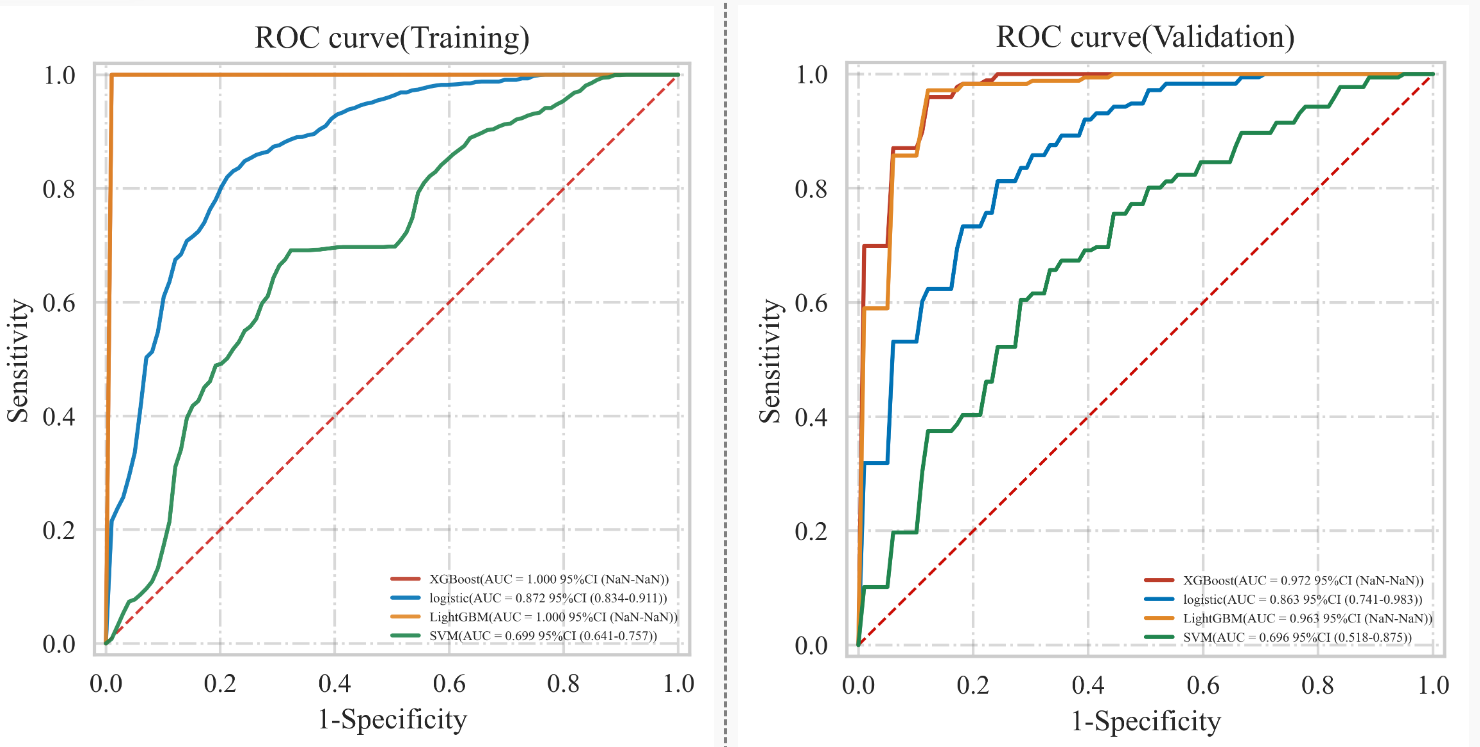


**Figure 2. ROC curve of multiple models**

Note: A. ROC curve in training set; B. ROC curve in the internal validation set.

**3.4 Model Performance Evaluation**

Testing the predictive ability of the XGBoost algorithm on the risk of heart failure in patients after CRA in the validation set. The results showed that the AUC of the XGBoost model in the validation set was **0.972**, the prediction accuracy was **0.921**, the sensitivity was **1.000**, the specificity was **0.892**, the positive predictive value was **0.911**, the negative predictive value was **0.931**, and the F1 score was **0.954**. See Figure3 for the Receiver operating characteristic of XGBoost model in the validation set.


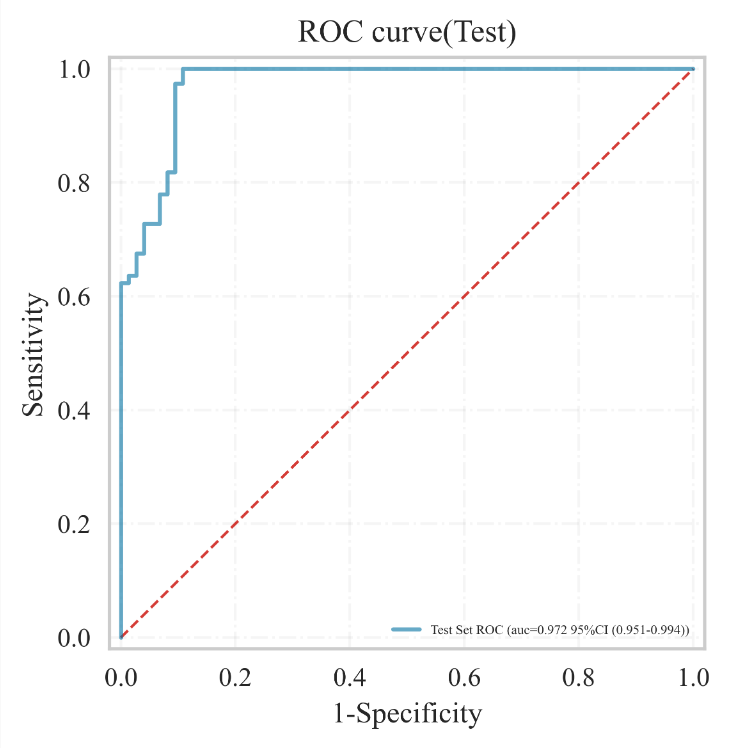


**Figure 3. XGBoost's ROC curve in the validation set**
